# Characterization of T cell immunity in chronic hepatitis B virus-infected mothers with postpartum alanine transaminase flare

**DOI:** 10.1186/s12879-021-06634-2

**Published:** 2021-09-06

**Authors:** Meiting Huang, Yunfei Gao, Xueru Yin, Xuelian Zhang, Yaohua Hao, Jing Hu, Zhihua Liu

**Affiliations:** 1 Department of Infectious Diseases, Nanfang Hospital, Southern Medical University, Guangzhou, 510515 China; 2 Present Address: Department of Infectious Diseases, Huizhou Third People’s Hospital, Guangzhou Medical University, Huizhou, 516002 China; 3 Present Address: Department of Obstetrics and Gynaecology, Zengcheng Branch of Nanfang Hospital, Southern Medical University, Guangzhou, 511340 China; 4 Department of Nosocomial Infection Administration, Zhujiang Hospital, Southern Medical University, Guangzhou, 510280 China; 5 Liver Diseases Center, Shenzhen Hospital of Southern Medical University, Shenzhen, 518000 China

**Keywords:** Hepatitis B virus, Postpartum, Alanine transaminase flare, T cell immunity

## Abstract

**Background:**

Postpartum alanine transaminase (ALT) flares occur frequently in chronic hepatitis B virus (HBV)-infected mothers with antepartum antiviral therapy (AVT). We aimed to characterize the T cell immunity in HBV-infected mothers experiencing postpartum ALT flares.

**Methods:**

Twenty HBV-infected pregnant women who received AVT at 26–28 weeks of gestation were enrolled and followed up until 15–18 weeks postpartum. Among the 20 HBV-infected pregnant women, 6 experienced postpartum ALT flare (AF mothers), while 14 did not (NAF mothers). T lymphocyte phenotypes and functions were analyzed using flow cytometry.

**Results:**

Compared to NAF mothers, the quantitative HBsAg levels in AF mothers decreased significantly at 6–8 or 15–18 weeks postpartum. Significant differences in HBeAg levels between these groups were only found at delivery. Regulatory T cell (Treg) numbers in AF mothers were lower than those of NAF mothers before AVT; however, there were no significant differences in Treg numbers at other follow-up points. Expression of other T cell phenotypes were similar between the two groups. T cells in AF mothers produced more pro-inflammatory cytokines (IFN-γ, IL-21, TNF-α, IL-2) or less anti-inflammatory cytokine (IL-10) than those in NAF mothers before, during, or after antiviral treatment. The ratio of IFN-γ to IL-10 producing by CD4^+^ T cells or CD8^+^ T cells was higher in AF mothers than that in NAF mothers during pregnancy or after delivery.

**Conclusions:**

The characteristics of T cell immunity was distinct between mothers with postpartum ALT flare and those without ALT flare from pregnancy to postpartum, which indicated that T cell immunity might get involved in postpartum ALT flare.

## Background

Globally, hepatitis B virus (HBV) infection continues to be a major public health problem. The World Health Organization (WHO) estimated that in 2015, 257 million people were living with chronic HBV infection, and that HBV-related diseases resulted in 887,000 deaths annually worldwide [1]. In highly endemic countries, mother-to-child transmission (MTCT) is the main route of HBV transmission; approximately 40–50% of HBsAg^+^ patients acquire infections via MTCT [2]. According to the latest WHO estimates, the proportion of children under 5 who are chronically infected with hepatitis B virus has decreased from 5% in the pre-vaccination period from the 1980s to the early 2000s to less than 1% in 2019 [1]. Reducing the incidence of MTCT is still a major challenge. Studies have shown that the levels of HBV DNA in HBV-infected mothers is an independent risk factor for MTCT [3–5]. It was reported that MTCT did not occur in mothers with a viral load < 6 log_10_ copies/mL [6]. Studies have shown that along with the administration of the HBV vaccine and immunoglobulin, antepartum antiviral therapy (AVT) can further reduce the incidence of MTCT in pregnant women with high viral loads [7–9]. Previous studies mainly focused on the effectiveness and safety of AVT, as well as the probability and severity of maternal alanine transaminase (ALT) flare after delivery [10]. The characteristics of postpartum ALT flare are mainly mild to moderate abnormalities, which can resolve spontaneously in the majority of mothers [11]. Currently, whether antiviral intervention during pregnancy increases the incidence of postpartum ALT flare remains controversial [7, 12, 13].

A successful pregnancy depends on the immune system of a pregnant woman, which not only needs to tolerate paternal antigens, but also needs to protect the woman and growing fetus against invading pathogens [14]. During the various stages of pregnancy, the immune system of pregnant women is dynamically changed. Pro-inflammatory immunity is dominant in the first trimester of pregnancy, while the second phase of pregnancy is predominantly anti-inflammatory. Pro-inflammatory immunity becomes dominant again in the last stage of pregnancy [15]. Studies have shown that regulatory T cell (Treg) frequency in the peripheral blood of pregnant women is decreased during pregnancy, which may be caused by changes in estrogen and progesterone levels [16]. This decrease may also occur due to maternal Tregs migrating to the maternal–fetal surface to prevent maternal–fetal rejection, resulting in decreased peripheral blood Treg frequency [17]. Therefore, the immune status of chronic HBV-infected pregnant women is different from that of chronic HBV-infected women who are not pregnant. These alterations reverse rapidly after delivery. Current hypotheses infer that alterations in the immune system during pregnancy might be responsible for a high rate of postpartum ALT flares in HBV-infected women [18]. However, immunological characteristics of HBV-infected mothers experiencing postpartum ALT flare has not been investigated.

This study aimed to characterize the T cell immunity of HBV-  infected mothers and explore the immunological mechanisms of postpartum ALT flare by comparing T cell immunity of HBV-infected mothers who experienced postpartum ALT flare with HBV-infected mothers without ALT flare.

## Patients and methods

### Patients

From September 2017 to April 2020, 20 pregnant women with chronic HBV infections who received prophylactic antiviral intervention (telbivudine or tenofovir) for the prevention of MTCT were enrolled and followed up at the Nanfang Hospital in Guangzhou, China. These pregnant women were in the phase of immune tolerance of chronic HBV infection at enrollment, showing HBeAg positivity, normal level of ALT, and high level of HBV DNA (≥ 6 log_10_ IU/mL) before antiviral intervention. All subjects recruited for this study were negative for hepatitis C virus and human immunodeficiency virus. Additionally, those receiving concurrent treatment involving cytotoxic drugs, immune modulators, glucocorticoids, as well as those with major systemic disease malignancies, liver cirrhosis, heart diseases, or renal dysfunctions were excluded.

Short-term AVT was administered to the subjects beginning at week 26–28 of gestation until delivery. They were visited at week 26–28 of gestation (before antiviral intervention), 1 month after antiviral intervention, at delivery, 6–8 weeks postpartum, and 15–18 weeks postpartum. ALT flare was defined as ≥ 2 × upper limit of normal (ULN). ALT flare mothers (AF mothers) were HBV-infected mothers experiencing a postpartum ALT flare (n = 6), while non-ALT flare mothers (NAF mothers) were HBV-infected mothers with postpartum ALT < 2 × ULN (n = 14).

### Virological and serological assessments

The levels of serum HBsAg and HBeAg were measured using a Roche Elecsys assay (Roche, Basel, Switzerland). Serum HBV DNA levels were quantitatively determined by real-time polymerase chain reaction (PCR) using a commercial nucleic acid diagnostic kit (Sansure Biotech, Changsha, China) with an lower limit of detection (LOD) of 100 IU/mL. Serological markers and HBV DNA assays were performed at the Laboratory of Viral Hepatitis Research, Nanfang Hospital (Guangzhou, China). The levels of serum ALT and aspartate transaminase (AST) were tested at the department of clinical laboratory of Nanfang Hospital (Guangzhou, China).

### Flow cytometric analysis of peripheral blood mononuclear cells (PBMCs)

Peripheral blood venous samples were obtained from individual women before, during, and after stopping antiviral intervention. PBMCs were isolated by Ficoll–Hypaque density gradient centrifugation and cryopreserved in liquid nitrogen for deferred analysis [19]. For flow cytometry analysis, thawed PBMCs were stained with Live/Dead Fixable dead cell stain (eBioscience, San Diego, CA, USA) for 10 min at room temperature. The cells were then stained for 30 min at 4 °C in the dark using the following antibodies (Abs): peridinin chlorophyll protein complex (PerCP)–Cy5.5- or fluorescein isothiocyanate (FITC)-anti-CD4 (clones SK3 and RAP-T4, respectively; BD Biosciences, San Jose, CA, USA), FITC- or allophycocyanin (APC)-anti-CD8 (clone RPA-T8; BD Biosciences, San Jose, CA, USA), phycoerythrin (PE)-anti-CD45RA (HI100; BD Biosciences, San Jose, CA, USA), PE–CY7-anti-CD62L (clone DREG-56; BD Biosciences, San Jose, CA, USA), PE–CY7-anti-CD25 (clone M-A251; BD Biosciences, San Jose, CA, USA), APC-anti-CD69 (clone FN50; Biolegend, San Diego, CA, USA), PE–CY7-anti-CD38 (clone HIT2; BioLegend, San Diego, CA, USA) and PE-anti-PD-1 (clone MIH4; BD Biosciences, San Jose, CA, USA). Intranuclear APC-anti-forkhead box P3 (FOXP3; clone PCH101; eBioscience, San Diego, CA, USA) staining was performed according to the manufacturer’s instructions. The stained cells were evaluated by flow cytometry using the BD FACSDiva system (BD Biosciences, San Jose, CA, USA), and the data were analyzed using FlowJo software (TreeStar Inc., Ashland, OR, USA) [20].

### Flow cytometric analysis of intracellular cytokine staining (ICS)

For analyzing intracellular cytokine production, 5 × 10^5^ cells/well were stimulated with 5 μg/mL of anti-CD3 monoclonal antibody (mAb; clone 145-2C11; BD Biosciences) and 5 μg/mL of soluble anti-CD28 mAb (clone 37.51; BD Biosciences, San Jose, CA, USA) in a 96-well flat-bottomed plate at 37 °C for 24 h. Brefeldin A (10 μg/mL; BD Biosciences, San Jose, CA, USA) was added during the last 6 h of incubation. The cells were stained with Live/Dead Fixable dead cell stain (eBioscience, San Diego, CA, USA) at room temperature. After being washed, the cells were stained with PerCP-Cy5.5-anti-CD4 and FITC-anti-CD8 at 4 °C for 30 min. The surface-stained cells were fixed and permeabilized using a fixing/permeabilizing reagent (BD Biosciences, San Jose, CA, USA) for 20 min, followed by staining with PE–Cy7-anti-interferon (IFN)-y (clone B27; BD Biosciences, San Jose, CA, USA), PE-anti-interleukin (IL)-10 (clone JES3-9D7; Biolegend, San Diego, CA, USA), PE-anti-IL-21 (clone 3A3-N2.1; BD Biosciences, San Jose, CA, USA), APC-anti-tumor necrosis factor (TNF)-α (clone 6401.1111; BD Biosciences, San Jose, CA, USA), PE-anti-IL-2 (clone MQ1-17H12; BD Biosciences, San Jose, CA, USA), and PE-Cy7-anti-IL-4 (clone 8D4-8; BD Biosciences San Jose, CA, USA) for 30 min. Analyses were performed using the BD FACSDiva system (BD Biosciences, San Jose, CA, USA) and the FlowJo program (TreeStar Inc., Ashland, OR, USA) [21].

### Statistical analysis

Data were expressed as either the median with a 95% confidence interval (CI) or an interquartile range. The non-parametric Mann–Whitney U test was used for statistical analysis based on two-tailed hypothesis tests using GraphPad Prism software (v.8.0; GraphPad Software, San Diego, CA, USA). P values < 0.05 were considered significant.

## Results

### Clinical characteristics of mothers with chronic HBV infection

The clinical characteristics are comparable between the AF mothers and the NAF mothers. No statistically significant differences were found between the two groups in terms of age, number of births, weeks of gestation, AVT duration, or delivery methods. The levels of ALT or AST in HBV-infected mothers from the AF group or NAF group were all within the normal range before antiviral intervention, and the median levels of ALT (interquartile range) were 22.00 (15.50, 23.50) and 21.00 (15.75, 24.25) U/L, respectively, while the median levels of AST (interquartile range) were 21.00 (16.50, 29.00) and 19.00 (16.75, 21.25) U/L, respectively, and no statistically significant differences were found. ALT and AST were within 1 × ULN during antiviral treatment in the NAF mothers (including one month after antiviral intervention and at delivery), while one mother from the AF mothers had ALT over 2 × ULN and her ALT levels returned to normal at delivery. Other AF mothers maintained ALT and AST levels in the normal range (1 × ULN) during antiviral intervention. At 6–8 weeks postpartum, 2 mothers (33.33%) from the AF group developed transient ALT > 2 × ULN. Another 4 mothers had ALT > 2 × ULN at 15–18 weeks postpartum. Two mothers from the AF group continued to have ALT abnormalities, and antiviral therapy was restarted after delivery (Table 1).

**Table 1:** Clinical characteristics of mothers with chronic HBV infection

	AF mothers (n = 6)	NAF mothers (n = 14)	P value
Age (years)	30.00 (25.25, 33.75)	27.00 (26.00, 29.00)	0.25
Gravidity	1.00 (1.00, 1.50)	1.00 (1.00, 2.00)	> 0.99
Parity	1.00 (1.00, 1.50)	1.00 (1.00, 1.25)	> 0.99
Delivery gestations (weeks)	39.00 (38.75, 39.25)	40.00 (38.00, 40.00)	0.5
Duration of AVT (days)	101.00 (91.00, 105.30)	95.00 (79.25, 105.80)	0.44
Type of delivery—no. (%)			
Vaginal	5 (83.33%)	11 (78.57%)	> 0.99
Caesarean section	1 (16.67%)	3 (21.43%)	
Before AVT (gestational 26–28 weeks)			
ALT(U/L)	22.00 (15.50, 23.50)	21.00 (15.75, 24.25)	0.7
AST(U/L)	21.00 (16.50, 29.00)	19.00 (16.75, 21.25)	0.54
Gestational 30–32 weeks			
ALT(U/L)	27.50 (16.00, 47.00)	16.00 (12.75, 25.25)	0.15
AST(U/L)	29.00 (22.75, 34.00)	20.59 (16.00, 22.00)	0.05
Delivery			
ALT(U/L)	17.50 (11.00, 24.50)	18.00 (13.00, 20.00)	0.94
AST(U/L)	22.00 (15.25, 25.25)	21.00 (16.25, 25.75)	0.98
Postpartum 6–8 weeks			
ALT(U/L)	38.00 (30.25, 98.50)	38.00 (18.75, 47.75)	0.41
ALT(n, > 2 × ULN)	2 (33.33%)	0	0.08
AST(U/L)	29.50 (19.00, 86.50)	25.50 (19.00, 36.00)	0.51
AST(n, > 2 × ULN)	2 (33.33%)	0	0.08
Postpartum 15–18 weeks			
ALT(U/L)	106.60 (40.00, 215.80)	28.00 (23.50, 46.00)	0.03
ALT(n, > 2 × ULN)	4 (66.67%)	0	0
AST(U/L)	57.00 (33.75, 158.50)	24.00 (21.00, 30.00)	0
AST(n, > 2 × ULN)	2 (33.33%)	0	0.08

Data are shown as median (*IQR* interquartile range)

*AVT* antepartum antiviral therapy; *ALT* alanine transaminase; *AST* aspartate aminotransferase

### Virological and serological characteristics of HBV-infected mothers during pregnancy and postpartum

**Fig. 1:**
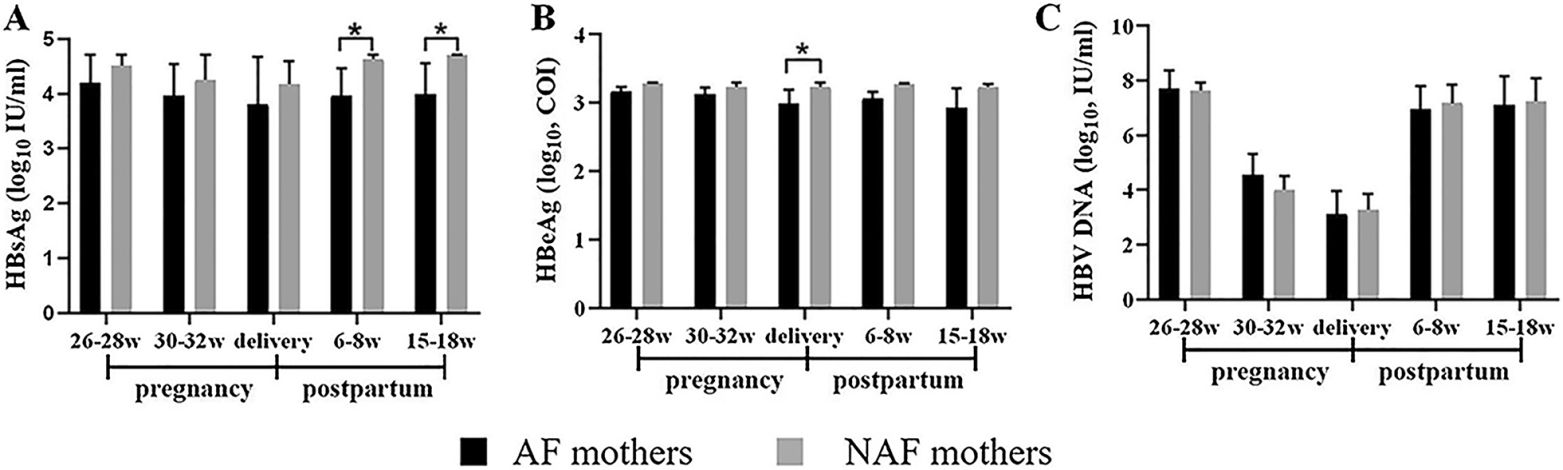
Compared the difference of the levels of HBsAg (**A**), HBeAg (**B**), or HBV DNA (**C**) at each follow-up point between the AF mothers (n = 6) and the NAF mothers (n = 14). *p < 0.05

The median levels of HBsAg in the AF mothers was lower than that of the NAF mothers before antiviral intervention, during AVT, or after antiviral treatment discontinuation postpartum. Significant differences were found only at 6–8 weeks and 15–18 weeks postpartum (p = 0.459, p = 0.294, p = 0.415, p = 0.027, p = 0.021). All mothers experienced a slight decline in the levels of HBsAg during AVT and increased levels after delivery (Fig. 1A). In addition, the levels of HBeAg in the AF group were lower than those in the NAF group at 5 follow-up points from 26 to 28 weeks of gestation to 15–18 weeks after birth, with a statistically significant difference between the two groups only occurring at delivery (p = 0.035) (Fig. 1B). With respect to HBV DNA levels, no significant difference was found between the AF and NAF mothers at any of the follow-up points (Fig. 1C).

### Comparison of the CD4^+^ T cell phenotypes of AF and NAF mothers before, during, and after AVT

**Fig. 2:**
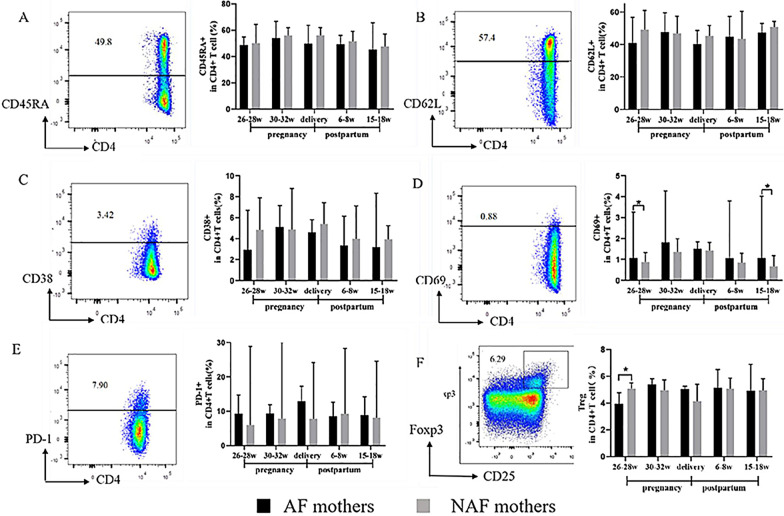
Characteristics of CD4^+^T cell phenotypes in AF mothers. **A**–**E** Expression of CD45RA, CD62L, CD38, CD69, PD-1 on CD4^+^ T cells. **F** Treg frequency in AF or NAF mothers. (AF mothers, *n* = 6; NAF mothers, *n* = 14). Data represent the median with 95% CI. *P < 0.05

To analyze the characteristics of CD4^+^ T cell phenotypes in AF mothers, we compared the differences in phenotype expression of CD4^+^ T cells based on the presence of CD45RA, CD62L, CD38, CD69, and PD-1 between the AF and NAF groups before, during, and after antiviral intervention (Fig. 2A–E). No significant differences were observed in the expression of CD45RA, CD62L, CD38, or PD-1 by CD4^+^ T cells at any of the follow-up points. However, we found that the activation marker CD69 was expressed by CD4^+^ T cells in AF mothers at a significantly higher rate than that in NAF mothers at 26–28 weeks of gestation (before antiviral intervention) and 15–18 weeks after birth (p = 0.04, p = 0.04) (Fig. 2D). We further examined the amount of Tregs (CD4^+^CD25^+^Foxp3^+^) between the two groups before, during, and after antiviral intervention and found that the number of Tregs in AF mothers was significantly higher than that in NAF mothers before antiviral intervention (i.e. at 26–28 weeks gestation) (p = 0.02). There were no significant differences in the amounts of Tregs between the two groups during and after stopping antiviral intervention (Fig. 2F).

### Comparison of CD8^+^T cell phenotypes of AF and the NAF mothers before, during, and after AVT

**Fig. 3:**
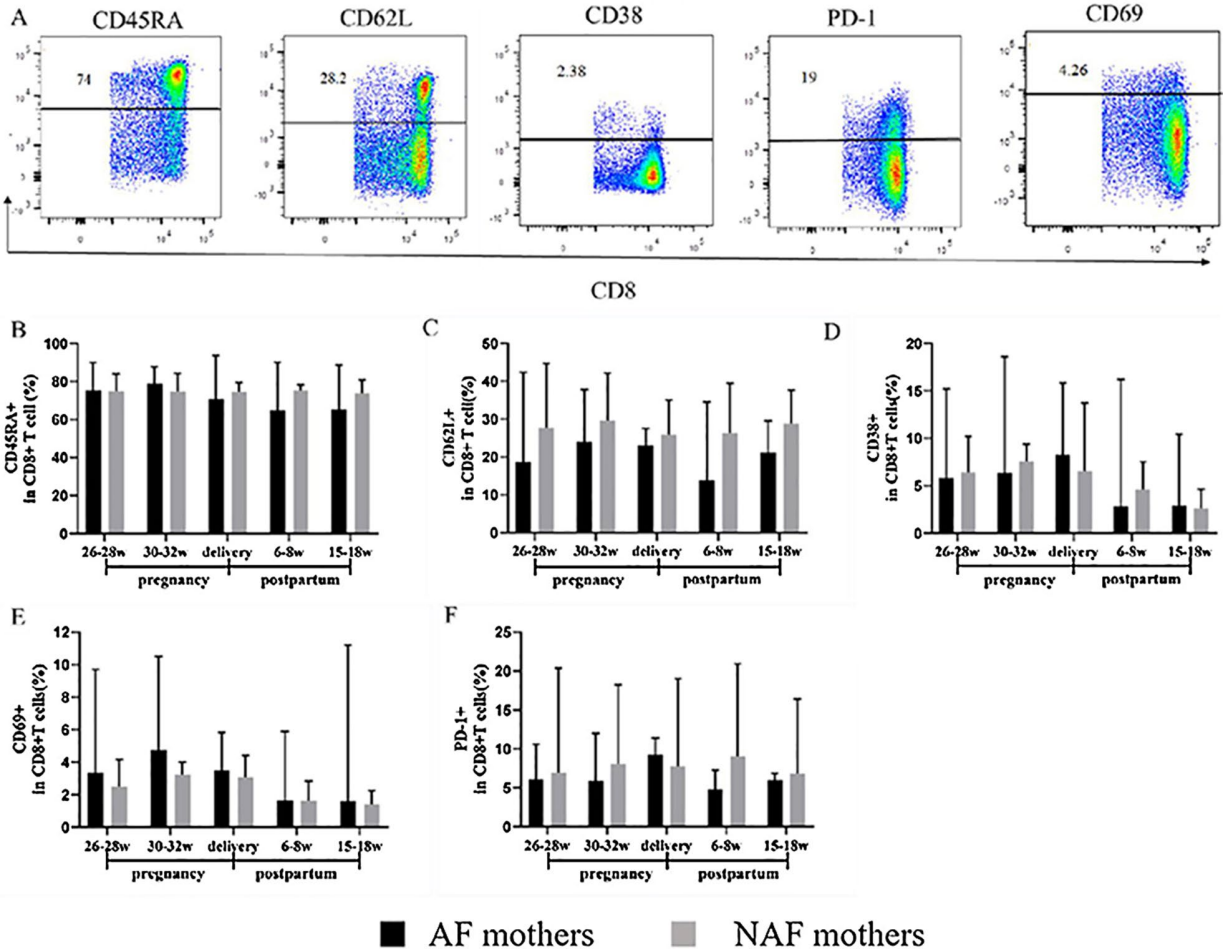
Characteristics of CD8^+^T cell phenotypes in AF mothers. **A** Representative FACS plots showing the phenotype expression of CD4^+^ T cells based on the presence of CD45RA, CD62L, CD38, CD69, and PD-1. **B**–**F** Expression of CD45RA, CD62L, CD38, CD69, and PD-1 by CD8^+^ T cells. (AF mothers, *n* = 6; NAF mothers, *n* = 14). Data represent the median with 95% CI. *P < 0.05

Representative FACS plots showing the phenotypes of CD8^+^ T cells based on the presence of CD45RA, CD62L, CD38, CD69, and PD-1 are shown in Fig. 3A. From 26 to 28 weeks gestation to 15–18 weeks after birth, the numbers of CD8^+^CD45RA^+^ cells, CD8^+^CD62L^+^ cells, CD8^+^CD38^+^ cells, CD8^+^CD69^+^ cells, and CD8^+^PD-1^+^ cells were similar between the AF and NAF mothers (Fig. 3B–F).

### CD4^+^ T cells in AF mothers produced more pro-inflammatory cytokines

**Fig. 4:**
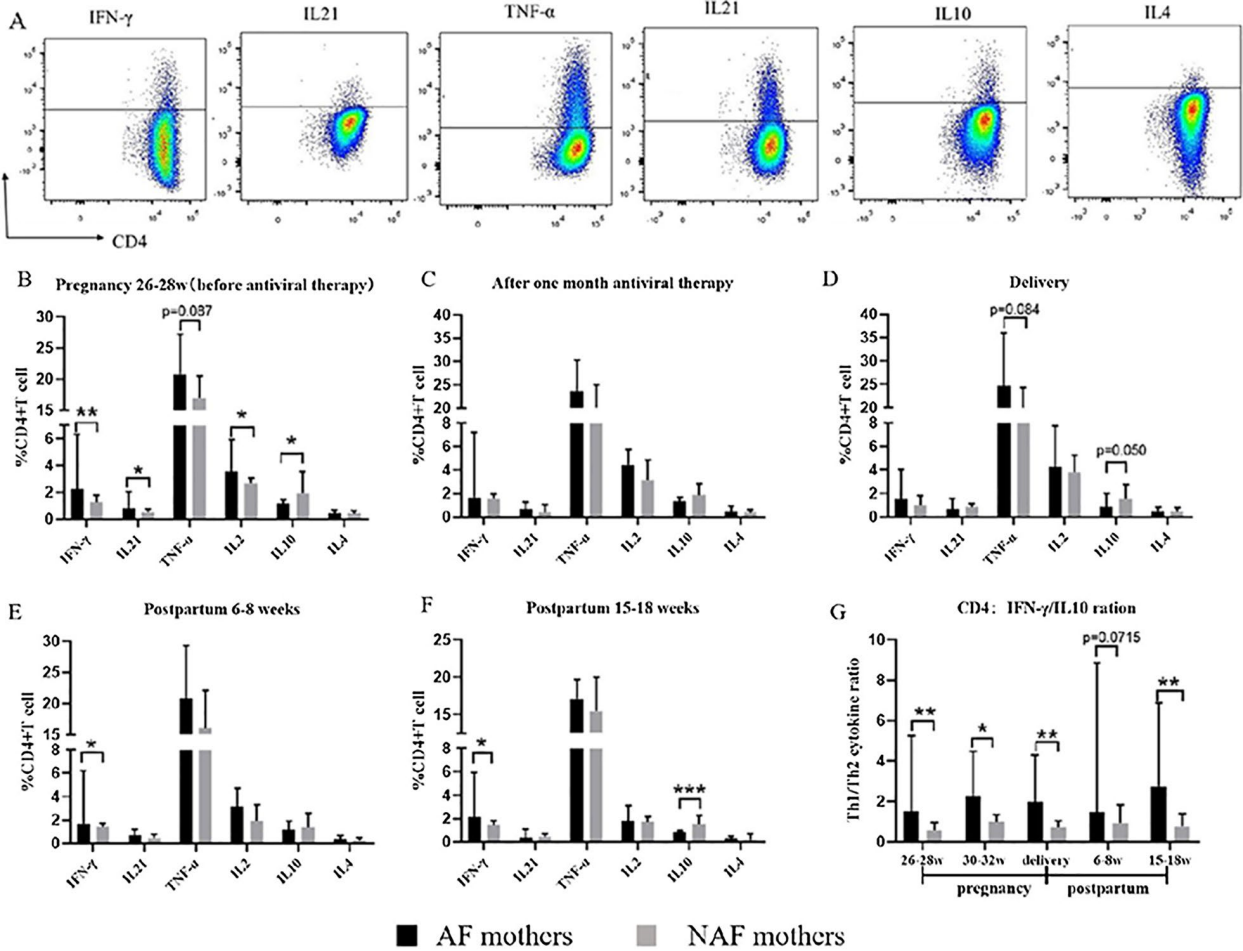
CD4^+^ T cells in AF mothers tend to secrete pro-inflammatory cytokines. **A** Representative dot plots of the production of cytokines (IFN-γ, IL-21, TNF-α, IL-2, IL-10 and IL-4) in CD4^+^ T cells. **B**–**F** Percentages of cytokine-producing CD4^+^ T cells in AF (*n* = 6) or NAF mothers (*n* = 14) at 26–28 weeks of gestation, 30–32 weeks of gestation, delivery, 6–8 weeks postpartum, or 15–18 weeks postpartum. **G** Data representing the ratio of pro-inflammatory cytokine to anti-inflammatory cytokine (IFN-γ:IL-10) in CD4^+^ T cells from 26 to 28 weeks of gestation to 15–18 weeks postpartum. The data represent the median with 95% CI. *P < 0.05, **P < 0.005

Following anti-CD3/anti-CD28 stimulation, we analyzed the levels of cytokines secreted by CD4^+^ T cells in 6 AF and 14 NAF mothers using flow cytometry. Representative FACS dot plots of cytokine production from CD4^+^ T cells are shown in Fig. 4A. Before antiviral treatment (at 26–28 weeks of gestation, Fig. 4B), CD4^+^ T cells in AF mothers secreted more IFN-γ, IL-21, and IL-2 than CD4^+^ T cells in NAF mothers (p = 0.007, p = 0.035, p = 0.021). Additionally, it appears that the secretion of TNF-α by CD4^+^ T cells was higher in AF mothers than that in NAF mothers (p = 0.087). However, compared to the CD4^+^ T cells from NAF mothers, the cells from AF mothers displayed a reduced ability to secrete anti-inflammatory cytokine IL-10 (p = 0.024). The secretion of IL-4 by CD4^+^ T cells was not significantly different between the two groups (p > 0.999) (Fig. 4B). During AVT (including one month after antiviral therapy and at delivery), there was no statistically significant difference in the secretion of various cytokines by CD4^+^ T cells between the AF and NAF mothers (Fig. 4C, D). After stopping antiviral intervention (including at 6–8 weeks postpartum and 15–18 weeks postpartum), the ability of CD4^+^ T cells to secrete IFN-γ was greater in AF mothers than that in NAF mothers (p = 0.049, p = 0.035). Moreover, AF mothers displayed attenuated IL-10 secretion at 15–18 weeks postpartum (p = 0.004) (Fig. 4E, F). Finally, when comparing the frequency ratio of IFN-γ-secreting CD4^+^ T cells to IL-10-secreting CD4^+^ T cells in AF mothers to that of NAF mothers at all follow-up points, we found that the ratio in AF mothers was significantly higher than that in NAF mothers (p = 0.002, p = 0.046, p = 0.001, p = 0.005) before antiviral intervention, one month after antiviral intervention, at delivery, and at 15–18 weeks postpartum. At 6–8 weeks postpartum, there was no significant difference in the ratio of pro-inflammatory/anti-inflammatory cytokines produced by CD4^+^T cells, but a similar trend was observed (p = 0.072) (Fig. 4G).

### CD8^+^ T cells from AF mothers produce more pro-inflammatory cytokines

**Fig. 5:**
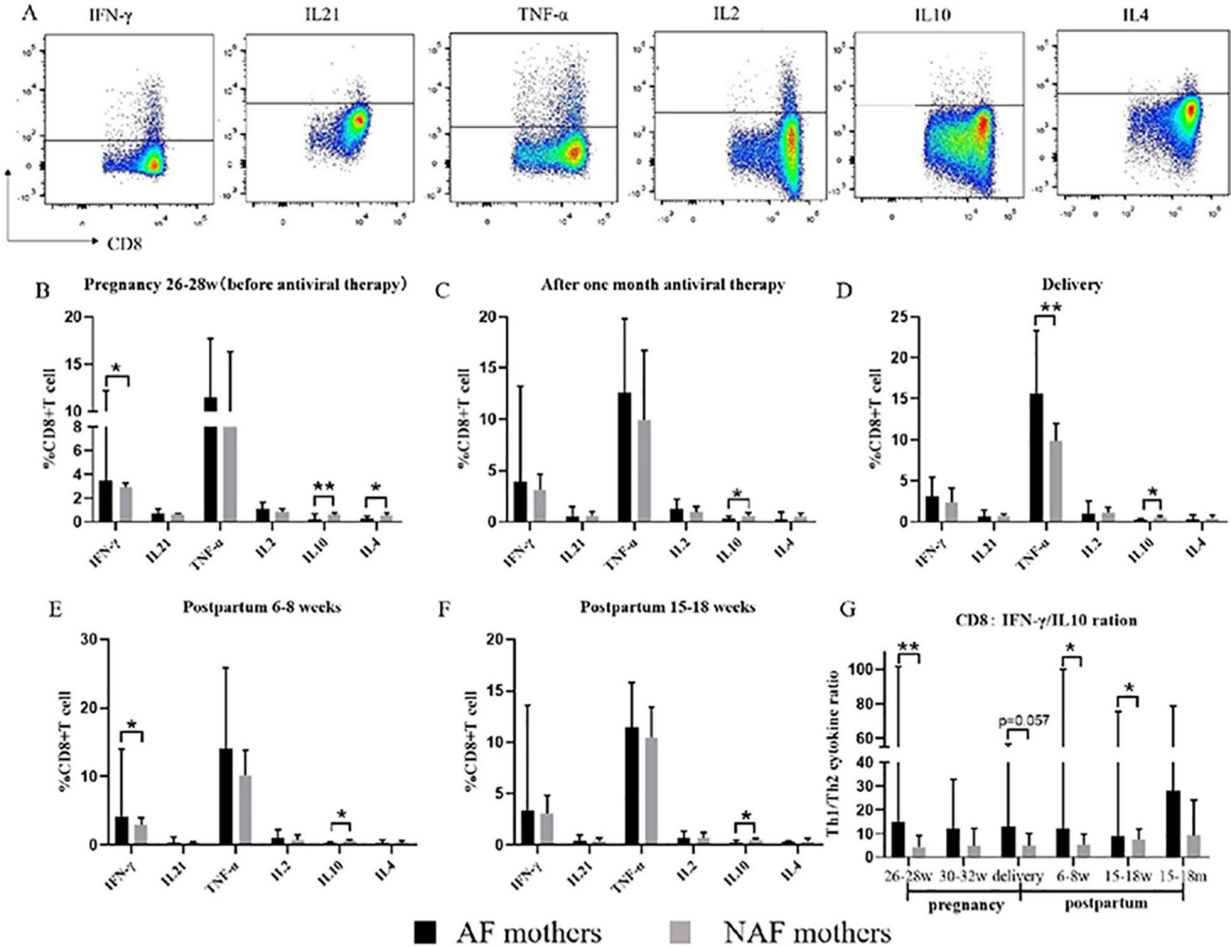
CD8^+^T cells in AF mothers tend to secrete pro-inflammatory cytokines. **A** Representative dot plots of the production of cytokines (IFN-γ, IL-21, TNF-α, IL-2, IL-10, and IL-4) in CD8^+^ T cells. **B**–**F** Percentages of cytokine-producing CD8^+^ T cells in AF mothers (*n* = 6) or NAF mothers (*n* = 14) at 26–28 weeks of gestation, 30–32 weeks of gestation, delivery, 6–8 weeks postpartum, or 15–18 weeks postpartum. **G** The data represent the ratio of pro-inflammatory cytokine to anti-inflammatory cytokine (IFN-γ:IL-10) in CD8^+^ T cells from 26 to 28 weeks of gestation to 15–18 weeks postpartum. The data represent the median with 95% CI. *P < 0.05, **P < 0.005

Representative FACS dot plots of pro-inflammatory cytokines (IFN-γ, IL-21, TNF-α, and IL-2) and anti-inflammatory cytokines (IL-4 and IL-10) produced by CD8^+^ T cells are shown in Fig. 5A. When comparing the pro-inflammatory and anti-inflammatory cytokine secretion from CD8^+^ T cells in the AF mothers with that of the NAF mothers before, during, and after AVT, we found that the amount of IFN-γ-secreting CD8^+^ T cells in AF mothers was significantly higher than that of NAF mothers at 26–28 weeks of gestation and at 6–8 weeks postpartum (p = 0.046, p = 0.035). The amount of IL-10-secreting CD8^+^ T cells in the NAF group was significantly higher than that in the AF group at all follow-up points (p = 0.005, p = 0.044, p = 0.039, p = 0.025, p = 0.002) (Fig. 5B–F). In addition, after analyzing the ratio of IFN-γ-secreting CD8^+^ T cells to IL-10-secreting CD8^+^ T cells at each follow-up point, the AF mothers were found to have a significantly higher ratio of these cells than that of the NAF mothers before and after stopping AVT (6–8 weeks postpartum and 15–18 weeks postpartum) (p = 0.007, p = 0.046, p = 0.027). The median ratio of the AF mothers was higher than that of the NAF mothers at 30–32 weeks of gestation or at delivery, but no significant difference was observed (p = 0.127, p = 0.058) (Fig. 5G). Briefly, the ability of CD8^+^ T cells to secrete pro-inflammatory factors in the AF group was higher than that in the NAF group, while the ability to secrete the anti-inflammatory factor IL-10 was significantly weaker than that in the NAF group.

## Discussion

In recent years, hepatitis B guidelines recommended that antiviral intervention should be administered to pregnant women with high HBV DNA levels to block MTCT and be withdrew after delivery [22–24]. It was reported that some of the HBV-infected mothers who underwent antiviral interventions in late pregnancy experienced postpartum ALT flares after drug withdrawal [25–28]. A large sample size, retrospective study showed that elevated ALT levels and detectable levels of HBV DNA at delivery were independent risk factors for postpartum disease flares [27]. However, there are limited data on T cell immunity in HBV-infected patients with postpartum ALT abnormalities. Thus, in the present study, we sought to examine T cell immunity of HBV-infected mothers who experienced postpartum ALT flare, with the aim of exploring immune mechanism which might underlie the postpartum ALT flares.

Our study found that 95% (19/20) of HBV-infected pregnant women maintained ALT normalization during the antiviral period. Postpartum ALT flares appeared in 30% (6/20) of HBsAg^+^/HBeAg^+^ mothers after stopping antiviral intervention, among which 4 cases spontaneously returned to normal and the other 2 cases required restarting antiviral treatment. We found that HBsAg levels in the AF mothers were significantly lower than those in the NAF mothers at 6–8 weeks and 15–18 weeks postpartum. This result showed that chronic HBV-infected mothers with lower HBsAg levels are prone to have ALT flare after delivery.

It has been reported that postpartum liver damage may occur due to changes in maternal immune status [11, 18]. In this study, we found that before antiviral treatment (at 26–28 weeks of gestation), compared to the Treg amounts in NAF mothers, those in AF mothers decreased, and T cells in AF mothers secreted significantly higher amounts of pro-inflammatory cytokines (IFN-γ, IL-21, IL-2) and less amounts of anti-inflammatory cytokines (IL-10). The ratio of pro-inflammatory cytokine (IFN-γ) to anti-inflammatory cytokine (IL-10) in CD4^+^ or CD8^+^ T cells was higher in AF mothers than that in NAF mothers before, during, or after AVT. Tregs are currently recognized as a CD4^+^ T cells that display significant immunosuppressive effects. The maintenance of immune tolerance is mainly manifested by Tregs having an immunosuppressive effect on HBV-specific T cell responses, leading to chronic infection [29, 30]. In addition, pro-inflammatory cytokines (such as IFN-γ and IL-12) prevent immune tolerance and eradicate virus, while anti-inflammatory cytokines (IL-10) maintain immune tolerance [31, 32].

Compared to NAF mothers, AF mothers had lower Treg frequency at 26–28 weeks of gestation, stronger pro-inflammatory immunity and weaker anti-inflammatory immunity from 26 to 28 weeks of gestation to 15–18 weeks postpartum. These results indicated that HBV-infected mothers had distinct characteristics of T cell immunity, which played an important role in postpartum ALT flares. Those mothers with lower Treg frequency and higher ratio of pro-inflammatory cytokine to anti-inflammatory cytokine in CD4^+^ or CD8^+^ T cells will be more likely to experience ALT flare postpartum. From above, we infer that when pro-inflammatory immunity is dominant (i.e., T cells secrete more pro-inflammatory cytokines and less anti-inflammatory cytokines; Treg amounts are decreased) in HBV-infected pregnant women, the patients’ immune tolerance tends to be attenuated, so that postpartum ALT flares are more likely to occur.

Because the differences in the profile of pro-inflammatory/anti-inflammatory cytokines existed as early as 26–28 weeks of gestation (before AVT), and remained to be significant until postpartum 15–18 weeks, we considered that distinct characteristics of T cell immunity was caused by neither AVT nor status of pregnancy. At baseline, the HBV-infected mothers exhibited distinct characteristics of T cell immunity, although their biochemical parameters were normal. Therefore, it is necessary to monitor ALT levels postpartum in chronic HBV-infected pregnant women and more attention should be paid to those with ALT flare because they are likely to experience the transition from immune tolerance phase to immune clearance phase.

This study was limited by the lack of a long-term follow-up analysis of HBsAg^+^/HBeAg^+^ mothers with high viral load who did not undergo AVT; consequently, we could not analyze whether AVT affected the incidence and severity of postpartum ALT flares. In addition, the sample size of this study was small, especially in AF group. More studies with larger sample size are needed to further validate the results of this study.

## Conclusion

In this study, we found that the characteristics of T cell immunity was distinct between AF and NAF mothers from pregnancy to postpartum. T cell immunity in AF mothers was characterized by lower Treg frequency and higher ratio of pro-inflammatory cytokine to anti-inflammatory cytokine in CD4+ or CD8+ T cells. The results indicated that maternal immune status might play an important role in postpartum ALT flare in HBV-infected mothers. Therefore, the HBV-infected mothers with ALT flare postpartum should be monitored closely because they might experience the transition from immune tolerance phase to immune active phase.

## Data Availability

The datasets used during the current study are available from the corresponding author upon reasonable request.

## Abbreviations

ALT: Alanine transaminase
HBV: Hepatitis B virus
AVT: Antepartum antiviral therapy
AF: ALT flare
NAF: Non-ALT flare
Treg: Regulatory T cell
WHO: The World Health Organization
MTCT: Mother-to-child transmission
ULN: The upper limit of normal
AST: Aspartate transaminase
PBMCs: Peripheral blood mononuclear cells
PerCP: Peridinin chlorophyll protein
FITC: Fluorescein isothiocyanate
APC: Allophycocyanin
PE: Phycoerythrin
FOXP3: Forkhead box P3
ICS: Intracellular cytokine staining
mAb: Monoclonal antibody
IFN: Interferon
IL: Interleukin
TNF: Tumor necrosis factor
CI: Confidence interval
